# Beyond biology: Social and geographic determinants of hypertension in rural Alabama communities

**DOI:** 10.1371/journal.pgph.0005835

**Published:** 2026-02-19

**Authors:** Sharlene D. Newman, D. Nichole Pompey, Linda Knol, Wanda Burton, Paige Johnson, Letisha Scott, Amie Brunson, In Gu Kang, Matthew Hudnall

**Affiliations:** 1 Alabama Life Research Institute, The University of Alabama, Tuscaloosa, Alabama, United States of America; 2 Department of Human Nutrition, Hospitality and Sport Management, The University of Alabama, Tuscaloosa, Alabama, United States of America; 3 Capstone College of Nursing, The University of Alabama, Tuscaloosa, Alabama, United States of America; 4 School of Social Work, The University of Alabama, Tuscaloosa, Alabama, United States of America; 5 Culverhouse Business School, The University of Alabama, Tuscaloosa, Alabama, United States of America; Tribhuvan University Institute of Medicine, NEPAL

## Abstract

Hypertension remains a leading modifiable risk factor for cardiovascular morbidity and mortality in the United States. Despite advances in detection and treatment, disparities in hypertension prevalence, control, and outcomes persist across racial, gender, and geographic lines, particularly in the rural Deep South. To examine the associations between race, gender, age, and geography on systolic and diastolic blood pressure among adult patients in West Alabama, and to identify patterns that may inform equitable, place-based interventions. De-identified health records from 3,462 adult patients across nine West Alabama counties were analyzed. Systolic and diastolic blood pressure were modeled as dependent variables using multivariate analyses including race, gender, age, and zip code. Interaction terms were examined to assess moderating effects between demographic and geographic variables. Systolic blood pressure (SBP) was most strongly predicted by age (*p* < 0.0001), with steeper increases among men. The steeper increase for men with age was driven by Black men – Black men showed a steeper SBP increase with age than Black women while White women showed a steeper increase than White men. Diastolic blood pressure (DBP) was independently associated with race, gender, and zip code (*p* < 0.05), with higher DBP among Black adults across geographic areas. Geographic variation persisted for both SBP and DBP, suggesting the influence of local social determinants of health. Distinct demographic and geographic patterns in blood pressure highlight the intersection of biological and structural factors driving cardiovascular disparities in rural Alabama. Addressing these inequities will require multilevel strategies integrating clinical care, community resources, and place-based policy interventions.

## Introduction

Hypertension remains one of the leading modifiable risk factors for cardiovascular morbidity and mortality in the United States. Despite overall advances in detection and management, disparities in the prevalence of hypertension, control, and outcomes remain across racial, gender, and geographic lines [1,2]. African American adults in particular experience earlier onset, higher prevalence, and lower rates of blood pressure control compared to White adults, contributing to disproportionately high rates of stroke, kidney disease, and cardiovascular mortality [3,4]. Additionally, rural Americans have higher odds of having hypertension than urban dwellers [5,6]. These inequities persist even when adjusting for income, education, and access to care [7], underscoring the potential role of structural racism in shaping cardiovascular outcomes [8,9].

Gender also plays an important role in the epidemiology of hypertension [10,11]. Men are more likely to develop elevated blood pressure earlier in life, whereas women often exhibit steeper increases in both systolic and diastolic pressures after midlife, coinciding with menopause and changes in vascular physiology [10]. These trajectories result in a narrowing of the gender gap in later life [2], with older women facing particularly high risks of hypertension-related cardiovascular complications. Gender disparities are further shaped by social roles, caregiving responsibilities, and health care access, which influence adherence to treatment and engagement in preventive care [12,13].

Geography and community context further modify hypertension risk and outcomes. Rural populations experience higher rates of hypertension and cardiovascular disease, driven by social determinants of health (SDoH) including provider shortages, limited access to specialty care, longer travel distances, and economic disadvantage [7,14,15]. In Alabama, particularly in the Black Belt region, these barriers intersect with persistent poverty, underfunded health infrastructure, and racial inequities in the built environment (housing, food access, water systems), producing some of the highest cardiovascular mortality rates in the nation [16]. Geographic variation in blood pressure outcomes suggests that neighborhood- and zip code–level factors are as critical as individual clinical predictors [17].

An additional distinction of clinical importance lies in the separate contributions of systolic and diastolic blood pressure. Systolic hypertension, which increases with arterial stiffness and age, is a stronger predictor of cardiovascular events in older adults, whereas diastolic hypertension has been linked to stroke and cardiovascular risk among younger populations [18]. Research suggests that systolic pressure is more strongly associated with age and gender, while diastolic disparities often reflect racial differences and exposure to environmental and psychosocial stressors [3]. This makes simultaneous examination of systolic and diastolic outcomes essential for identifying nuanced disparities across populations.

Although many studies have documented hypertension disparities, fewer have examined how systolic and diastolic pressures vary simultaneously across race, gender, and geography in large, real-world datasets that capture diverse patients across health systems. Health information exchanges (HIEs) provide a unique opportunity to evaluate blood pressure outcomes at scale, integrating data from multiple providers and payers to reflect community-level patterns. Leveraging HIE data allows for a more comprehensive understanding of how demographic and geographic variables interact to shape cardiovascular risk, particularly in underserved rural areas.

This study focused on counties in West Alabama. Alabama has consistently been found to have a high prevalence of hypertension and a low control rate [19,20]. Data from the Alabama state health information exchange was used to examine differences in systolic and diastolic blood pressure by race, gender, age, and residential zip code in nine West Alabama counties. By distinguishing between systolic and diastolic pressures and incorporating geographic variation, the analysis provides a nuanced perspective on how biological, social, and structural factors converge to produce disparities in hypertension outcomes among adults in West Alabama.

## Methods

### Study design and data source

This study is part of a larger project designed to better understand and address factors that may underlie the high rate of hypertension in West Alabama, specifically 9 West Alabama counties (see Table 1). A retrospective analysis of blood pressure data from Alabama’s Health Information Exchange (HIE) from these nine counties from April 1, 2025 to May 9, 2025 was conducted. The authors obtained minimally identifiable protected health information, limited to patient ZIP codes. All data were transferred to a secure server and stored in a HIPAA-compliant environment, with access restricted to research team members approved by the Institutional Review Board (IRB). The sample included single clinic visits of 3,462 adult participants with available demographic and clinical information, including race, gender, age, residential zip code, and blood pressure values. All analyses were conducted using SAS 9.4, with significance defined at alpha level 0.05.

**Table 1:** Demographics of Target Area.

	Population	%Black	Median Income	%poverty	Without insurance
Alabama	5,024,279	25.7%	53,913	16.1%	9.9%
Bibb	22,915	19.7%	$41,770	12.6%	13%
Fayette	17,241	10.5%	$28,539	17.9%	13.8%
Greene	9,974	80.6%	$19,819	34.3%	11.6%
Hale	17,185	56.2%	$25,807	26.9%	11.4%
Lamar	13,972	10.2%	$43,324	17%	12.1%
Perry	8,317	67.8%	$27,057	33.7%	12.4%
Pickens	18,615	40.1%	$23,896	21.1%	12.5%
Sumter	11,853	70.9%	$27,099	35.1%	12.1%
Tuscaloosa	235,668	32.9%	$57,508	14.7%	10.3%

Data from 2020 Census

### Ethics statement

The study was approved by the University of Alabama IRB (# 23-08-6857). A waiver of written informed consent was obtained from the IRB.

### Blood pressure classification

Blood pressure stage was defined using established clinical cut points as suggested by the American Heart Association:

- Normal-elevated blood pressure: systolic <130 mmHg and diastolic <80 mmHg
- Stage 1 hypertension: systolic 130–139 mmHg or diastolic 80–89 mmHg
- Stage 2 hypertension: systolic 140–180 mmHg or diastolic 90–120 mmHg
- Hypertensive crisis: systolic >180 mmHg or diastolic >120 mmHg

### Descriptive analyses

Frequencies of categorical variables (race, gender, blood pressure stage, and zip code) were summarized. Frequencies of blood pressure stages were examined overall and stratified by race and gender. Continuous variables (age, systolic blood pressure, and diastolic blood pressure) were summarized and descriptive statistics including sample size, mean, standard deviation, median, minimum, and maximum were produced. Group-specific means were calculated separately by race and by gender.

### Normality assessment

The distributions of age, systolic blood pressure, and diastolic blood pressure were evaluated, stratified by race and gender. Tests of normality indicated that continuous outcomes were not normally distributed. Although analysis of variance (ANOVA) assumes normally distributed residuals, ANOVA is generally robust to moderate violations of this assumption in the presence of large sample sizes and reasonably balanced groups. Given the total sample size (n = 3,462) and analytic objectives, ANOVA was retained as the primary inferential method.

### Inferential analyses

Two separate general linear models (GLM) were estimated to evaluate predictors of systolic and diastolic blood pressure. Independent variables included race, gender, age (continuous), and patient residential zip code. Zip code was specified as a categorical control variable to account for geographic variation. The following interaction terms were included in both models: race × gender, race × age, race × zip code, gender × age, and gender × zip code. For interactions involving continuous age, slope analyses were performed to evaluate whether the relationship between age and blood pressure differed across demographic subgroups. Specifically, estimates were computed for separate regression slopes of age on systolic blood pressure and diastolic blood pressure within levels of gender and within racial groups. These slope estimates allowed assessment of whether systolic and diastolic disparities disproportionately varied by gender or race across the age span. For interactions involving geographic variation (by zip code), least squares means (LS-means) estimates were generated. Specifically, LS-means were used to evaluate differences in systolic blood pressure across zip codes within subgroups defined by race and gender, and to evaluate differences in diastolic blood pressure across counties within racial groups. Pairwise differences of LS-means were requested to further explore significant interaction effects.

### Multi-factor ANOVA of Systolic and diastolic blood pressure

Separate multi-factor ANOVA models were created to determine effects of gender, age, and zip code on systolic and diastolic blood pressure and whether those effects are independent or dependent on one (or more) of the other factor(s).

## Results

### Blood pressure summary

There are 3,462 individuals included in the analysis data; of this total 2131 are African American (62%), 1331 are Caucasian (38%), 895 are male (26%), and 2567 are female (74%). Normal blood pressure has 1142 (33%), Stage 1 hypertension has the most individuals with 1197 (35%), Stage 2 hypertension has 1044 (30%), and hypertensive crisis has the least with 79 (2%). Table 2 illustrates the overall demographic summary statistics for race, gender, blood pressure stage, and blood pressure (systolic and diastolic).

**Table 2:** Summary Table of Age, Systolic and Diastolic Blood Pressure, and Blood Pressure Stage Overall and by Gender and Race.

Data Statistic	Overall N = 3462	Female N = 2567	Male N = 895	White N = 1331	Black N = 2131
Age (years)					
Mean (SD)	53.02 (17.9)	51.51 (18.4)	57.36 (15.7)	52.60 (17.6)	53.28 (18.1)
Median	55	53	60	54	55
Min, Max	18, 103	18, 103	18, 101	18, 103	18, 101
Systolic					
Mean (SD)	131.18 (20.0)	130.20 (19.9)	134.00 (20.1)	126.98 (20.1)	133.81 (19.5)
Median	130	128	132	125	132
Min, Max	73, 225	73, 216	82, 225	73, 216	80, 225
Diastolic					
Mean (SD)	80.29 (11.3)	79.99 (11.4)	81.17 (11.0)	78.53 (11.0)	81.39 (11.4)
Median	80	80	81	78	81
Min, Max	41, 148	41, 148	48, 132	41, 148	42, 132
Blood Pressure Stage					
Normal	1142 (33%)	888 (35%)	254 (28%)	534 (40%)	608 (29%)
Stage 1	1197 (35%)	882 (34%)	315 (35%)	457 (34%)	740 (35%)
Stage 2	1044 (30%)	745 (29%)	299 (33%)	317 (24%)	727 (34%)
Hypertensive Crisis	79 (2%)	52 (2%)	27 (3%)	23 (2%)	56 (3%)

### Systolic Blood Pressure (SBP)

The results indicate statistically significant effects of race, age, and geographic area (zip code) on SBP. Age and race emerged as the strongest predictors (both *p* < 0.0001), while zip code also showed significant geographic variation (*p* = 0.0328). Moreover, racial differences in blood pressure varied by zip code (*p* = 0.0294), and the relationship between age and SBP differed between males and females (*p* = 0.0244). In addition, gender differences in blood pressure depended on geographic area (*p* < 0.0001). A complete summary of model estimates is presented in Table 3.

**Table 3:** Type III SS Summary of Multi-Factor ANOVA of Systolic Blood Pressure.

Source	DF	F Value	Pr > F
Race	1	17.96	**<.0001**
Gender	1	0.93	0.3348
Age	1	161.44	**<.0001**
Zip Code	71	1.34	**0.0328**
Race*Zip Code	42	1.46	**0.0294**
Age*Gender	1	5.07	**0.0244**
Gender*Zip Code	58	1.88	**<.0001**

**Fig 1:**
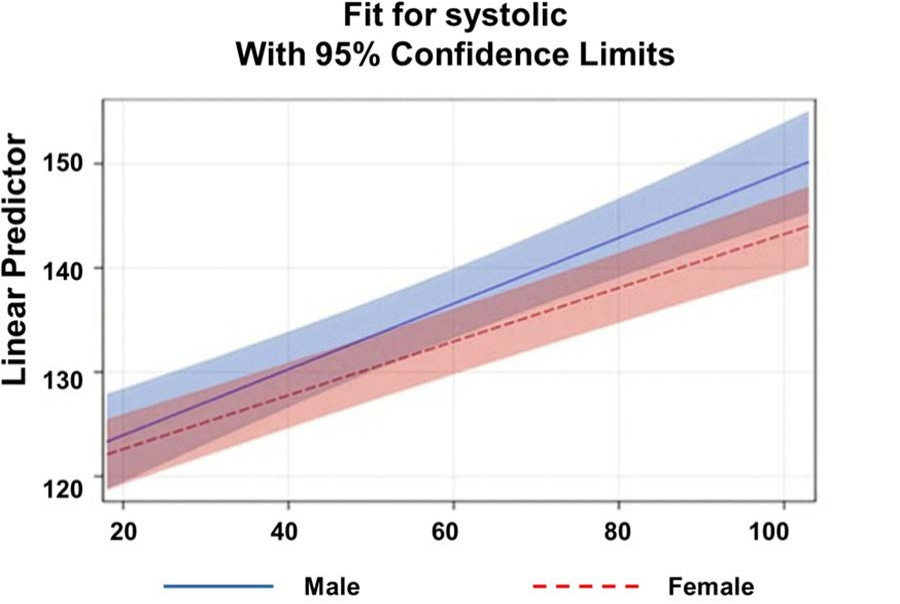
Linear Prediction of Systolic Blood Pressure Across Age by Gender.

Systolic blood pressure increased markedly with age, though the rate of increase differed by gender. Fig 1 presents predicted SBP as a function of age, stratified by gender. Both men and women showed significant positive associations between age and SBP, with steady increases across the lifespan. The estimated slope of age was 0.36 mmHg per year for men and 0.30 mmHg per year for women, indicating a steeper increase among men. At younger ages, men had slightly higher predicted SBP, and this difference widened with advancing age. The 95% confidence bands around each regression line demonstrate that the age effect on SBP is robust in both groups, with partial overlap between genders. Overall, these findings confirm that systolic blood pressure rises with age for both sexes, however, unlike the previous literature the rate of increase is greater in males.

**Fig 2:**
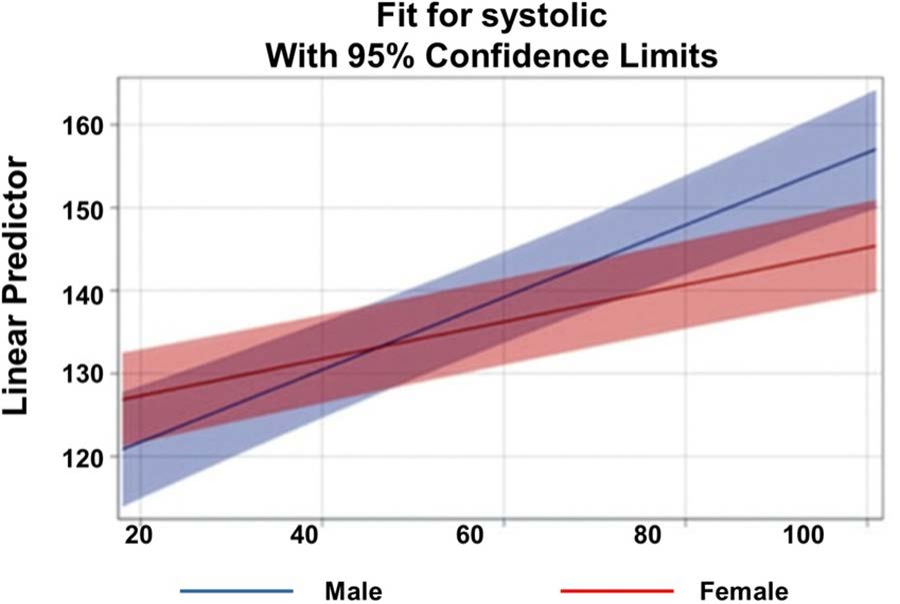
Linear Prediction of Systolic Blood Pressure Across Age by Gender for Race = Black.

**Fig 3:**
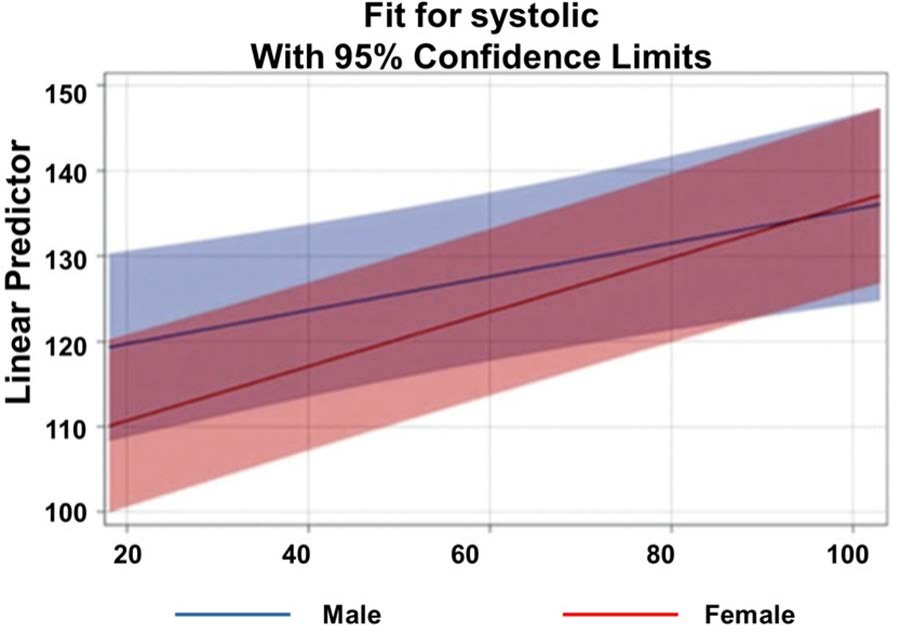
Linear Prediction of Systolic Blood Pressure Across Age by Gender for Race = White.

Race was also a significant predictor, and the interaction between race, age, and gender was further examined. Figs 2 and 3 illustrate the age-by-gender slopes for Black and White participants, respectively. Among Black participants, the estimated slope for age was β = 0.46 for males and β = 0.34 for females. At younger ages, Black females had higher predicted SBP than Black males; however, the steeper male slope led to convergence and eventual crossover around age 45, with males surpassing females thereafter, contradicting previously reported gender differences. Among White participants, the estimated slope for age was β = 0.22 for males and β = 0.26 for females. White males exhibited higher SBP at younger ages, but because White females’ slope was steeper, their predicted SBP converged with and surpassed that of males around age 90. This steeper slope for White females aligns with prior literature indicating that women have lower SBP at younger ages but experience a more rapid increase with aging.

### Diastolic Blood Pressure (DBP)

The results indicate statistically significant effects of race, gender, age, and geographic area (zip code) on diastolic blood pressure. Race, age, and gender were strong, independent predictors of DBP after adjustment for all other variables (*p* < 0.0001). Geographic area was also a significant factor, with notable differences across zip codes (*p* = 0.0127). Furthermore, racial differences in DBP varied by geographic area, as indicated by a significant race × zip code interaction (*p* = 0.0048). A full summary of the model estimates is provided in Table 4.

**Table 4:** Type III SS Summary of Multi-Factor ANOVA of Diastolic Blood Pressure.

Source	DF	F Value	Pr > F
Race	1	18.20	**<.0001**
Gender	1	19.05	**<.0001**
Age	1	63.06	**<.0001**
Zip Code	71	1.42	**0.0127**
Race*Zip	42	1.66	**0.0048**

Diastolic blood pressure was significantly influenced by race, gender, age, and geographic location. Race remained an independent predictor, and the magnitude of racial differences varied across zip codes. Geographic location itself contributed meaningfully to variability in DBP, underscoring the importance of contextual environmental factors.

Follow-up (slicing) analyses revealed that DBP varied significantly across zip codes for both Black (*p* < 0.0001) and White (*p* = 0.0342) participants. To further characterize these differences, adjusted mean DBP values were compared across zip codes. Among Black participants, the adjusted means ranged from the high 60s to high 90s mmHg, with some zip codes exhibiting markedly elevated estimates. Wider confidence intervals in sparsely populated zip codes suggested greater uncertainty in those areas. Among White participants, adjusted mean DBP values ranged from the low 60s to high 80s mmHg, consistently lower than those observed among Black participants across all geographic areas.

While many pairwise differences between zip codes were not statistically significant, the overall race × zip code interaction confirmed significant variability in diastolic blood pressure across geographic areas for both racial groups. These findings provide evidence of both intra- and inter-racial variability in DBP that is strongly influenced by local geographic context.

## Discussion

This analysis of blood pressure data from more than 3,462 patients in West Alabama reveals meaningful disparities in blood pressure outcomes across race, gender, age, and geography. Importantly, the findings underscore distinct patterns for systolic versus diastolic blood pressure, suggesting that these measures capture different physiological mechanisms and risk profiles. Across all age groups, Black participants exhibited higher mean SBP and DBP compared to White participants. SBP exhibited significant gender differences, whereas DBP was shaped by both race and gender effects.

Systolic blood pressure was most strongly predicted by age, consistent with extensive evidence that systolic hypertension becomes increasingly prevalent with arterial stiffening and vascular aging [21]. Among White participants, SBP followed the previously reported pattern, with women exhibiting greater increases with age than men. This has important clinical implications given that elevated systolic pressure is a well-established predictor of myocardial infarction, stroke, and overall cardiovascular morbidity and mortality in older adults [22]. Moreover, Ji and colleagues [23] demonstrated that women experience increased cardiovascular risk even at lower systolic thresholds than men, underscoring the importance sex-specific blood pressure monitoring and intervention strategies.

Contrary to prior work indicating that women experience sharper increases in SBP with age [10], the present data suggest a steeper trajectory among men overall that is driven by Black patients. One explanation for the previous finding is that women show a steeper age-related increase in SBP due to hormonal changes [10]. Indeed, Black women do experience increases in SBP with age, and their slope is steeper than that of White women, corroborating Richardson and Brown’s [24] findings that race and gender may have a multiplicative effect on hypertension risk. However, Black patients demonstrated a markedly different trajectory than White patients: younger Black women had higher SBP than their male counterparts, but Black men showed a steeper rise in SBP with advancing age. Prior research has linked low socioeconomic position in both childhood and adulthood to higher blood pressure [25], particularly among Black men [26]. In a previous study examining perceived stress and depression among Black residents of the Alabama Black Belt, we found that men reported increasing stress, anxiety, and depression with age, whereas women did not [27]. This pattern suggests that psychosocial stressors may contribute disproportionately to age-related increases in SBP among Black men and could help explain the observed gender differences in hypertension risk.

By contrast, diastolic blood pressure was independently predicted by race, with Black adults exhibiting significantly higher DBP values even after controlling for age, gender, and geographic area. This pattern echoes prior findings from Carnethon et al. [3], who documented persistent racial disparities in hypertension despite adjustments for socioeconomic and clinical covariates. The independent association between race and DBP may reflect the cumulative physiological consequences of chronic psychosocial stress, structural racism, and adverse neighborhood conditions - factors that have been shown to increase vascular resistance and elevate diastolic pressure [28,29]. McEvoy et al. [30] also reported that isolated diastolic hypertension remains a significant predictor of cardiovascular disease in younger adults, underscoring the long-term implications of early disparities.

Geographic variation across zip codes further highlights the powerful influence of social determinants of health (SDoH). Both systolic and diastolic pressures varied significantly by location, suggesting that contextual factors such as socioeconomic deprivation, limited health care infrastructure, food insecurity, and transportation barriers play a key role in shaping cardiovascular risk [31,32]. These findings align with previous work demonstrating that neighborhood-level socioeconomic disadvantage is associated with poorer blood pressure control and worse cardiovascular outcomes [17]. In West Alabama, where rural communities continue to experience underinvestment in health services and high poverty rates [33], the geographic disparities observed here underscore the urgency of place-based strategies for hypertension control.

Combined census data from the nine West Alabama counties included in this analysis—Bibb, Fayette, Greene, Hale, Lamar, Perry, Pickens, Sumter, and Tuscaloosa—provide important socioeconomic context. Across these counties, the median household income is approximately $50,000, with a per capita income of $24,000. The overall poverty rate is 19%, and roughly 22% of residents rely on the Supplemental Nutrition Assistance Program (SNAP). A GINI index (a summary measure of income inequality) of 0.42 reflects moderate income inequality, while labor force participation (57%) and unemployment (4.8%) further underscore the region’s economic challenges. Considerable variation exists by zip code: for example, areas with the lowest prevalence of stage 1 and 2 hypertension were located in Tuscaloosa County (e.g., zip code 35475, median income $104,852), whereas those with the highest hypertension prevalence had substantially lower median incomes (e.g., zip code 36744 in Perry County, $33,983). These socioeconomic disparities highlight the broader context of structural disadvantage that likely contributes to elevated cardiovascular risk across the region.

### Limitations

Several limitations should be acknowledged. Socioeconomic and behavioral factors (e.g., diet, weight, physical activity, medication adherence) were not uniformly available, limiting the ability to fully adjust for all risk factors. Additionally, geographic patterns were analyzed at the zip code level, which may obscure important within-community variation. The analysis focused on only two races. There is a growing Hispanic population that should be included in future studies. Finally, the data were derived from a health information exchange (HIE) system, which may be subject to variability in measurement practices and incomplete data capture across sites. Although the majority of federally qualified health centers in the state are connected to the HIE, participation is voluntary and not all private or smaller rural clinics contribute data. Consequently, the sample may be biased toward lower-income patients who more commonly receive care in safety-net settings. The relatively small sample size likely reflects several structural factors. Alabama does not mandate HIE participation for healthcare providers, and adoption remains limited among smaller rural clinics. In addition, with the exception of Tuscaloosa County, the included counties are sparsely populated and characterized by limited healthcare access, further constraining data availability. Finally, the HIE did not consistently capture information on anti-hypertensive medication use, limiting the ability to fully account for treatment effects.

## Conclusions

aken together, these findings suggest that strategies to reduce hypertension disparities must address both biological aging processes and the structural inequities that shape exposure to risk. Clinically, systolic blood pressure appears most sensitive to age and gender, whereas diastolic pressure disproportionately affects Black adults across the lifespan. We emphasize that observed differences between Black and White adults are not hypothesized to reflect biological differences. Rather, we posit that these disparities are driven by differential exposure to chronic and cumulative toxic stress, shaped by social and environmental conditions. Public health interventions should prioritize rural and underserved areas, where geographic variation in blood pressure reflects broader inequities in access to care, housing stability, transportation, and access to foods and beverages that promote health.

The use of HIE data in this study demonstrates the value of population-level surveillance for identifying nuanced disparities that might be missed in smaller clinical samples. By integrating race, gender, and geography, the analysis highlights the need for multilevel interventions - from clinical management and prevention programs to structural and community-based efforts targeting the social determinants of cardiovascular health. Strengthening rural health infrastructure, expanding access to preventive care, and empowering community-based organizations in West Alabama represent critical next steps toward reducing the disproportionate cardiovascular burden experienced by Black communities and by women in later life.

## References

[1] Martin SS, Aday AW, Almarzooq ZI, Anderson CAM, Arora P, Avery CL, et al. 2024 Heart Disease and Stroke Statistics: A Report of US and Global Data From the American Heart Association. Circulation. 2024;149(8):e347–913. doi: 10.1161/CIR.0000000000001209 38264914 PMC12146881

[2] Tsao CW, Aday AW, Almarzooq ZI, Anderson CAM, Arora P, Avery CL, et al. Heart Disease and Stroke Statistics-2023 Update: A Report From the American Heart Association. Circulation. 2023;147(8):e93–621. doi: 10.1161/CIR.0000000000001123 36695182 PMC12135016

[3] Dainty KN, Colquitt B, Bhanji F, Hunt EA, Jefkins T, Leary M, et al. Understanding the Importance of the Lay Responder Experience in Out-of-Hospital Cardiac Arrest: A Scientific Statement From the American Heart Association. Circulation. 2022;145(17):e852–67. doi: 10.1161/CIR.0000000000001054 35306832

[4] Akinyelure OP, Jaeger BC, Moore TL, Hubbard D, Oparil S, Howard VJ, et al. Racial Differences in Blood Pressure Control Following Stroke: The REGARDS Study. Stroke. 2021;52(12):3944–52. doi: 10.1161/STROKEAHA.120.033108 34470498 PMC10032619

[5] Heindl B, Howard G, Clarkson S, Kamin Mukaz D, Lackland D, Muntner P, et al. Urban-rural differences in hypertension prevalence, blood pressure control, and systolic blood pressure levels. J Hum Hypertens. 2023;37(12):1112–8. doi: 10.1038/s41371-023-00842-w 37407675

[6] Kuehn BM. Hypertension Rates in Rural Areas Outpace Those in Urban Locales. JAMA. 2020;323(24):2454. doi: 10.1001/jama.2020.9382 32573675

[7] Commodore-Mensah Y, Turkson-Ocran RA, Cooper LA, Himmelfarb CD, Reilly CM. The role of social determinants of health in hypertension management in Black adults: a review. Curr Hypertens Rep. 2021;23:68. doi: 10.1007/s11906-021-01159-4

[8] Albert MA, Williams DR, Cooper LA. Structural racism, social determinants, and cardiovascular health equity. Circulation. 2024;149:20–32. doi: 10.1161/CIRCULATIONAHA.123.065432

[9] Churchwell K, Elkind MSV, Benjamin RM, Carson AP, Chang EK, Lawrence W, et al. Call to Action: Structural Racism as a Fundamental Driver of Health Disparities: A Presidential Advisory From the American Heart Association. Circulation. 2020;142(24):e454–68. doi: 10.1161/CIR.0000000000000936 33170755

[10] Drury ER, Wu J, Gigliotti JC, Le TH. Sex differences in blood pressure regulation and hypertension: renal, hemodynamic, and hormonal mechanisms. Physiol Rev. 2024;104(1):199–251. doi: 10.1152/physrev.00041.2022 37477622 PMC11281816

[11] Yeo W-J, Abraham R, Surapaneni AL, Schlosser P, Ballew SH, Ozkan B, et al. Sex Differences in Hypertension and Its Management Throughout Life. Hypertension. 2024;81(11):2263–74. doi: 10.1161/HYPERTENSIONAHA.124.22980 39229711 PMC11483212

[12] Graff TC, Birmingham WC, Wadsworth LL, Hung M. Doing it all: Effects of Family Responsibilities and Marital Relationship Quality on Mothers’ Ambulatory Blood Pressure. Ann Behav Med. 2024;58(1):67–78. doi: 10.1093/abm/kaad058 37824850 PMC10729791

[13] King AC, Oka RK, Young DR. Ambulatory blood pressure and heart rate responses to the stress of work and caregiving in older women. J Gerontol. 1994;49(6):M239-45. doi: 10.1093/geronj/49.6.m239 7963275

[14] Malla G, Long DL, Cherrington A, Goyal P, Guo B, Safford MM, et al. Neighborhood Disadvantage and Risk of Heart Failure: The Reasons for Geographic and Racial Differences in Stroke (REGARDS) Study. Circ Cardiovasc Qual Outcomes. 2024;17(3):e009867. doi: 10.1161/CIRCOUTCOMES.123.009867 38328917 PMC10950536

[15] Metlock FE, Hinneh T, Benjasirisan C, Alharthi A, Ogungbe O, Turkson-Ocran R-AN, et al. Impact of Social Determinants of Health on Hypertension Outcomes: A Systematic Review. Hypertension. 2024;81(8):1675–700. doi: 10.1161/HYPERTENSIONAHA.123.22571 38887955 PMC12166636

[16] Alabama Department of Public Health. Alabama Cardiovascular Disease Burden Report. Montgomery, AL: ADPH. 2020.

[17] Akinyelure O, Huang H, McKee CM. Neighborhood socioeconomic context and hypertension control in the rural South. Prev Chronic Dis. 2024;21:E45. doi: 10.5888/pcd21.230321

[18] Shelbaya K, Arthur V, Yang Y, Dorbala P, Buckley L, Claggett B, et al. Large-Scale Proteomics Identifies Novel Biomarkers and Circulating Risk Factors for Aortic Stenosis. J Am Coll Cardiol. 2024;83(5):577–91. doi: 10.1016/j.jacc.2023.11.021 38296402 PMC12582517

[19] He S, Park S, Fujii Y, Pierce SL, Kraus EM, Wall HK, et al. State-Level Hypertension Prevalence and Control Among Adults in the U.S. Am J Prev Med. 2024;66(1):46–54. doi: 10.1016/j.amepre.2023.09.010 37877903 PMC10898652

[20] Hsiao TW, Fede L, Gocke C, Waller LA, Ali MK, Varghese JS. County Prevalence, Awareness, and Control of High Blood Pressure From Health Kiosks in the United States, 2017-2024. Hypertension. 2026;83(2):e25829. doi: 10.1161/HYPERTENSIONAHA.125.25829 41410032

[21] Izzo JL Jr, Levy D, Black HR. Clinical Advisory Statement. Importance of systolic blood pressure in older Americans. Hypertension. 2000;35(5):1021–4. doi: 10.1161/01.hyp.35.5.1021 10818056

[22] Psaty BM, Furberg CD, Kuller LH, Cushman M, Savage PJ, Levine D, et al. Association between blood pressure level and the risk of myocardial infarction, stroke, and total mortality: the cardiovascular health study. Arch Intern Med. 2001;161(9):1183–92. doi: 10.1001/archinte.161.9.1183 11343441

[23] Ji H, Niiranen TJ, Cheng S. Sex differences in blood pressure and cardiovascular outcomes. Circ Res. 2021;128:1392–410. doi: 10.1161/CIRCULATIONAHA.120.04936 PMC7884079 33587655

[24] Richardson LJ, Brown TH. (En)gendering Racial Disparities in Health Trajectories: A Life Course and Intersectional Analysis. SSM Popul Health. 2016;2:425–35. doi: 10.1016/j.ssmph.2016.04.011 28111630 PMC5240637

[25] Walsemann KM, Goosby BJ, Farr D. Life course SES and cardiovascular risk: Heterogeneity across race/ethnicity and gender. Soc Sci Med. 2016;152:147–55. doi: 10.1016/j.socscimed.2016.01.038 26854625 PMC4792096

[26] James SA, Van Hoewyk J, Belli RF, Strogatz DS, Williams DR, Raghunathan TE. Life-course socioeconomic position and hypertension in African American men: the Pitt County Study. Am J Public Health. 2006;96(5):812–7. doi: 10.2105/AJPH.2005.076158 16571689 PMC1470586

[27] Newman SD, Newman MM. An examination of the association between perceived stress and autistic traits in a rural predominately African American community sample. Curr Psychol. 2023;43(7):6672–7. doi: 10.1007/s12144-023-04851-3

[28] Matthews KA, Cottington EM, Talbott E, Kuller LH, Siegel JM. Stressful work conditions and diastolic blood pressure among blue collar factory workers. Am J Epidemiol. 1987;126(2):280–91. doi: 10.1093/aje/126.2.280 3605056

[29] Kamarck TW, Schwartz JE, Shiffman S, Muldoon MF, Sutton-Tyrrell K, Janicki DL. Psychosocial stress and cardiovascular risk: what is the role of daily experience?. J Pers. 2005;73(6):1749–74. doi: 10.1111/j.0022-3506.2005.00365.x 16274452

[30] McEvoy JW, Yang W-Y, Thijs L, Zhang Z-Y, Melgarejo JD, Boggia J, et al. Isolated Diastolic Hypertension in the IDACO Study: An Age-Stratified Analysis Using 24-Hour Ambulatory Blood Pressure Measurements. Hypertension. 2021;78(5):1222–31. doi: 10.1161/HYPERTENSIONAHA.121.17766 34601965 PMC8516806

[31] Diez Roux AV. Neighborhoods and Health: What Do We Know? What Should We Do?. Am J Public Health. 2016;106(3):430–1. doi: 10.2105/AJPH.2016.303064 26885960 PMC4815954

[32] Mujahid MS, Diez Roux AV, Morenoff JD, Raghunathan TE, Cooper RS, Ni H, et al. Neighborhood characteristics and hypertension. Epidemiology. 2008;19(4):590–8. doi: 10.1097/EDE.0b013e3181772cb2 18480733

[33] Alabama Department of Public Health. Health Disparities in Alabama: A County-Level Overview. Montgomery, AL: ADPH. 2023.

